# The Efficacy of Li-ESWT Combined With VED in Diabetic ED Patients Unresponsive to PDE5is: A Single-Center, Randomized Clinical Trial

**DOI:** 10.3389/fendo.2022.937958

**Published:** 2022-06-23

**Authors:** Rongzhen Tao, Jianhuai Chen, Dujian Wang, Yunpeng Li, Jun Xiang, Lei Xiong, Junbiao Ji, Jie Wu, Shuang Zhou, Chunping Jia, Jianlin Lv, Jie Yang, Qinglai Tang

**Affiliations:** ^1^ Department of Urology, The Affiliated Jiangning Hospital of Nanjing Medical University, Nanjing, China; ^2^ Department of Andrology, Jiangsu Province Hospital of Chinese Medicine, Affiliated Hospital of Nanjing University of Chinese Medicine, Nanjing, China; ^3^ Department of Ultrasound, The Affiliated Jiangning Hospital of Nanjing Medical University, Nanjing, China; ^4^ Department of Urology, Jiangsu Provincial People’s Hospital, First Affiliated Hospital of Nanjing Medical University, Nanjing, China; ^5^ Department of Urology, People’s Hospital of Xinjiang Kizilsu Kirgiz Autonomous Prefecture, Xinjiang, China

**Keywords:** erectile dysfunction, diabetes mellitus, low intensity extracorporeal shock wave treatment, vacuum erectile device, phosphodiesterase type 5-inhibitors

## Abstract

**Introduction:**

Phosphodiesterase type 5-inhibitors (PDE5is) are the first-line treatment for patients with diabetes mellitus-induced erectile dysfunction (DMED), however, some patients are non-responser to PDE5is. We performed a perspective, randomized, comparative study to explore the efficacy of low intensity extracorporeal shock wave treatment (Li-ESWT) combined with vacuum erectile device (VED) in the treatment of DMED patients who were unresponsive to PDE5is.

**Methods:**

One hundred and five eligible patients were randomly divided into three groups: group A (VED), group B (Li-ESWT) and group C (VED plus Li-ESWT). Follow-up was conducted at 4 weeks, 8 weeks and 12 weeks after the end of treatment. The erectile function was estimated by the international index of erectile function-erectile function domain (IIEF-EF), erection hardness score (EHS), sexual encounter profile questions 2 and 3 (SEP2 and SEP3) and global assessment question 1 and 2 (GAQ1 and GAQ2) before and after treatment. The changes of five points in IIEF-EF were calculated as the minimal clinical important difference (MCID), which was considered as the main index of efficacy.

**Results:**

The MCID was achieved in 14.7%, 14.7% and 17.6% patients in group A at the follow up on 4 weeks, 8 weeks and 12 weeks, respectively (36.4%, 39.4% and 36.4% in group B; 36.4%, 51.5%, and 66.7% in group C). There were significant differences in the percentage of MCID cases between group A and group C at the follow up on 12 weeks (*P*<0.001), as well as that between group B and group C (*P*=0.014). Additionally, comparison in MCID within group C showed that there were significant differences between 4 weeks and 12 weeks follow-up (*P*=0.014).

**Conclusion:**

Our findings indicated the combined therapy Li-ESWT and VED was more beneficial to shift turn PDE5is non-responders to responders for moderate patients with DMED than VED or Li-ESWT monotherapy. Moreover, this study provided evidence that patients with DMED who failed after taking oral PDE5is could attempt to opt for an alternative physicotherapy (Li-ESWT or VED) prior to more invasive alternatives.

## Introduction

Diabetes mellitus (DM) is a common disease with a relative high prevalence of 9-11% (1, 2). One-third of patients have a microvascular complication at the time of diagnosis of diabetes, while more than half of male patients with diabetes will eventually develop ED and the treatment rate of ED in younger men with type 2 diabetes is up to four times higher than those without diabetes (3, 4). Phosphodiesterase type 5-inhibitors (PDE5is) are the first-line for these patients. However, clinical studies on effectiveness of oral PDE5i are mainly aimed at patients with mild to moderate diabetes mellitus-induced erectile dysfunction (DMED) (5, 6). Moderate and severe diabetic ED patients who are non-responser to PDE5is have to choose other options or PDE5is combined with novel emerging therapies (7).

In recent years, low intensity extracorporeal shock wave treatment (Li-ESWT), as a strongly-recommended option by increasing experts from various countries in the world, has been becoming a promising and encouraging physical modality, according to its satisfactory efficacy and safety, especially for ED patients with vascular factor (8–10). One double-blind, sham controlled study demonstrated that penile low intensity shock wave treatment was able to shift PDE5is non-responders to responders (11). Nevertheless, the other study on elevating long time effect of Li-ESWT found that the diabetic patients with severe ED who were initially successful had lost the effect of LI-ESWT during two-year follow-up (12). Meanwhile, diabetic patients with moderate to severe ED who were PDE5is non-responders, might be necessary to be received comprehensive management protocol or implantation of penile prosthesis (IPP) to obtain long-term efficacy (13).

Vacuum erectile device (VED), as yet, is simple, reversible and effective second-line therapeutic strategy for patients with PDE5i refused or failed, as well as for diabetic ED patients. Vacuum with a mechanical pump can enlarge penis, maintain penile length, get a non-physiological erection and augment an erection even in difficult-to-treat population (14, 15). Although intracavernosal and transurethal alprostadil is also effective in diabetic patients with ED of mixed aetiology (16, 17), however, the second-line therapeutic management is not usually accepted by patients and/or their sexual partners as a long-term therapeutic measure due to common adverse events such as burning, erythema, pain sensations from patients and vaginal burning or itching from sexual partners. VED could offer a viable alternative to intracavernosal injection, transurethral suppositories or topical administration of vasoactive agents (18).

Based on current conditions that most patients with mild DMED are effective with PDE5is, and those who are ineffective often have significantly improved symptoms after PDE5is combined with Li-EWST or VCD, while non-surgical treatment invalid patients with refractory and severe DMED usually have to receive IPP if they intend to the ideal curative effects, in spite of relatively significant postoperative pain (19). Therefore, initial PDE5is non-response patients with moderate DMED were selected as subjects of this study, and we performed a perspective, randomized, comparative study to explore whether Li-ESWT combined with VED was more effective than Li-ESWT or VED monothepy in the treatment of PDE5is non-responser with moderate DMED.

## Materials and Methods

### Subjects

The subjects of this study were diagnosed with DMED (T2DM) in the urology and andrology clinic of the Affiliated Jiangning Hospital of Nanjing Medical University from October 2019 to September 2021. Their medical history was more than six months and all of them were non-responders after using the maximum tolerated dose of PDE5is along with adequate sexual stimulation for more than 6 times (20). Total of 105 subjects were finally eligible. All patients were randomly divided into three groups with 35 cases in each group: group A (VED), group B (Li-ESWT) and group C (VED plus Li-ESWT).

Inclusive criteria: (1) patients with DMED (T2DM), aged between 20-65; (2) IIEF-EF: 11-16 scores; (3) fixed sexual partner maintaining a normal sexual relationship and trying sexual behavior at least once a week from the beginning to the end of the study; and (4) normal reproductive hormone, and erection hardness score (EHS) ≤ 2 and peak systolic velocity (PSV) < 25cm/s 10-15 min after the intracavernous injection of 10 ug prostaglandin E1 (PGE1).

Exclusive criteria: (1) severe diabetic complications were excluded, such as neuropathy, nephropathy, and retinopathy; (2) mental and psychological diseases, serious cardiovascular (including hypertension) and cerebrovascular diseases, liver and kidney dysfunction, malignant tumors, alcohol dependence and abnormal coagulation function; (3) hepatitis B/hepatitis C/HIV infection, spinal cord injury, genitourinary tract injury, inflammation, and external genital malformations; (4) ED patients with other organic or endocrine factors such as severe thyroid disease, end-stage renal failure, non-diabetic related metabolic diseases (including dyslipidemia), sleep disorders and other systemic diseases; (5) history of ED related surgery or treatment, such as radical prostatectomy, pelvic radiologic therapy; (6) bleeding disorders and those on anticoagulation therapy.

The age, duration of disorder, body mass index (BMI), IIEF-EF (baseline), EHS (baseline), testosterone, and PSV (baseline) of penile cavernous artery measured by color duplex doppler ultrasound (CDDU) were evaluated in each group of this study. In addition, this study was performed in accordance with the ethical standards laid down in the 1964 Declaration of Helsinki and its later amendments and approved by local ethical committee (No. 2019-03-026-k01). All patients gave their informed consent to the collection of clinical data in a prospectively maintained database and to the use of these data for research purposes.

### Schedule and Protocol

VED: Patients in group A and C were treatment with penile vacuum erectile device (Osbon, Timm Medical Technologies, Eden Prairie, MN, USA). Subjects were made sure to practiced how to use VED successfully by personal tutoring and watching an instructional video before enrollment of the study. Each treatment time was 15 minutes during 9-week trail period, which include repeatedly creating penile erection by pumping gradually and becoming penile softness by releasing vacuum, without the use of tension ring, 3 times a week.

Li-ESWT: Patients in group B and C were treated with electromagnetic type Li-ESWT (HD. ESWO-I, 80mm diameter, focusing probe, Shenzhen Hyde Medical Equipment Co., Ltd. Shenzhen, China). These patients were treated twice a week. After 3 weeks of treatment, they were intermittently treated for 3 weeks and then treated for 3 weeks, a total of 12 times. The treatment parameter was set under the shock pressure 7.5KV and pulse frequency 100 times/min, and the position of treatment was located in the distal, body and crura of each left and right side of penile cavernous body. Each site was impacted 300-400 times, a total of 1800-2400 times.

Li-ESWT plus VED: The treatment protocol of this group integrated that of the above two groups, and the interval between two treatments was necessary to be more than 24 hours.

All patients who participated in the studies were not allowed to receive PDE5is 1 month before and during the study. After the last treatment, they were allowed to consume PDE5is on demand. Schedule and protocol of the study was shown in **Supplementary Figure 1**.

### Follow-Up and Assessment of Therapeutic Efficacy

All subjects were assessed at 4 weeks, 8 weeks and 12 weeks after the end of treatment. The efficacy was measured by IIEF-EF, EHS, sexual encounter profile question 2 and 3 (SEP2 and SEP3), Global Assessment Question 1 and 2 (GAQ1 and GAQ2). Effectiveness at 4th, 8th, 12th week follow-up was determined by the score changes of IIEF-EF from baseline according to the minimal clinical important difference (MCID) (21) i.e. an increase of at least 5 points for moderate ED. Mean EHS level and per patient percentage of “yes” responses to SEP2 (successful penetration), SEP3 (successful intercourse), GAQ1 (improving erectile function) and GAQ2 (improving the ability to engage in sexual activity) were investigated as treatment outcomes.

### Statistical Analyses

All statistical analyses were performed using SPSS version 22.0 (SPSS Inc., Chicago, IL, USA) software. Firstly, the Shapiro-WilK test was used to test the normality of the initial descriptive data of continuous variables, which were expressed as mean ± SD or median (25%,75% quantile), and were compared using t-test or Mann-Whitney U test, as appropriate. Proportions were expressed as absolute numbers and percentages and compared using the Chi-squared test or Wilcoxon rank sum test as appropriate. The one-way analysis of variance (ANOVA) was performed to compare the mean IIEF-EF score changes with respect to baseline in subgroups A, B, and C. *P*<0.05 was taken to indicate statistical significance. Z-test was used for testing two proportions the with unpooled variance and the power was computed using the normal approximation method by PASS 15.0.5 (NCSS, Kaysville, Utah, USA).

## Results

### Demographic and Clinical Characteristics of Patients

One hundred patients completed the clinical trial and obtained all data (group A: 34 cases, group B: 33 cases and group C: 33 cases). Baseline characteristics of patients with DMED in three groups were shown in **Table 1**. There were no significant differences in the age, course of ED, BMI, testosterone level, IIEF-EF scores, EHS and PSV values of penile cavernous artery before treatment (*P*>0.05).

**Table 1:** Baseline characteristics of patients with diabetic erectile dysfunction in three groups.

Parameters	Group A (n = 34)	Group B (n = 33)	Group C (n = 33)	F Value	P Value
Age (mean ± SD, yr)	47.97 ± 5.69	46.70 ± 4.93	48.30 ± 3.49	1.032	0.360
ED Duration (mean ± SD, m)	45.53 ± 21.95	43.88 ± 27.16	45.27 ± 25.06	0.043	0.958
BMI (mean ± SD, points)	23.11 ± 5.99	23.33 ± 4.84	23.99 ± 3.36	0.296	0.744
Baseline PSV (mean ± SD, cm/s)	16.03 ± 2.05	15.86 ± 2.03	15.94 ± 2.36	0.050	0.497
Testosterone (mean ± SD, nmol/l)	15.29 ± 2.74	15.35 ± 2.46	14.85 ± 2.19	0.398	0.436
IIEF-EF (score)	13.38 ± 1.71	13.48 ± 1.62	13.30 ± 1.61	0.101	0.904
EHS (score)	1.82 ± 0.39	1.85 ± 0.36	1.82 ± 0.39	0.060	0.942

The data was analyzed by one-way analysis of variance (ANOVA) with a significance level alpha=0.05. ED, erectile dysfunction; BMI, body mass index; PSV, peak systolic velocity of penile artery; IIEF-EF, international index of erectile function erectile function domain; EHS, erection hardness score.

### Comparison of Efficacy Among and Within the Three Groups

The parameters of therapeutic efficacy included the proportion of cases achieving MCID (improving in IIEF-EF score is more than 5 score), the proportion of patients reporting successful penetration (SEP2), the proportion of patients reporting successful intercourse (SEP3), the proportion of cases improving erectile function (GAQ1), and the proportion of cases improving the ability to engage in sexual activity (GAQ2). The difference of parameters of therapeutic efficacy among three groups and within each group at all follow-up time points were shown in **Table 2**.

**Table 2:** The differences of parameters of therapeutic efficacy among three groups and within each group at various follow-up points.

Parameters	Follow-up	Group A	Group B	Group C	Chi-square value	P value
MCID (yes%,n)	4w	14.7%, 5	36.4%, 12	36.4%, 12	5.112	0.078
	8w	14.7%, 5	39.4%, 13	51.5%, 17	10.392	0.006*
	12w	17.6%, 6	36.4%, 12	66.7%, 22	17.038	<0.001*
	Chi-square value	0.148	0.086	6.066		
	P value	0.929	0.958	0.048*		
SEP2 (yes%,n)	4w	29.4%, 10	45.5%, 15	42.4%, 14	2.054	0.358
	8w	26.5%, 9	42.4%, 14	57.6%, 19	6.655	0.036*
	12w	29.4%, 10	39.4%, 13	66.7%, 22	10.016	0.007*
	Chi-square value	0.096	0.248	4.009		
	P value	0.953	0.883	0.135		
SEP3 (yes%,n)	4w	8.8%, 3	18.2%, 6	27.3%, 9	12.786	0.002*
	8w	8.8%, 3	18.2%, 6	21.2%, 7	2.087	0.352
	12w	8.8%, 3	21.2%, 7	24.2%, 8	3.042	0.219
	Chi-square value	–	0.130	0.330		
	P value	–	0.937	0.848		
GAQ1 (yes%,n)	4w	35.3%, 12	51.5%, 17	45.5%, 15	1.831	0.400
	8w	32.4%, 11	45.5%, 15	63.6%, 21	6.626	0.036*
	12w	35.3%, 12	45.5%, 15	66.7%, 22	6.843	0.033*
	Chi-square value	0.087	0.324	3.580		
	P value	0.957	0.850	0.167		
GAQ2 (yes%,n)	4w	14.7%, 5	27.3%, 9	36.4%, 12	3.830	0.147
	8w	14.7%, 5	36.4%, 12	36.4%, 12	5.112	0.078
	12w	14.7%, 5	36.4%, 12	39.4%, 13	5.810	0.055
	Chi-square value	–	0.818	0.086		
	P value	–	0.664	0.958		

The proportions were expressed as percentages and compared using the Chi-squared test. MCID(yes%): The percentage of patients meeting MCID≥ 5 score; SEP2(yes%): The percentage of patients reporting successful penetration; SEP3(yes%): The percentage of patients reporting successful intercourse; GAQ1 (improving erectile function), GAQ2 (improving the ability to engage in sexual activity). *P<0.05, there were statistically significant difference.

MCID, a change of 5 IIEF-EF points for moderate ED, was considered as the main index of efficacy, MCID in group A was achieved in 14.7%, 14.7%, and 17.6% of patients at the follow up on 4, 8, and 12 weeks, respectively. In group B, MCID was achieved in 36.4.6%, 39.4%, and 36.4% of patients at the follow up on 4, 8, and 12 weeks, respectively. In group C, MCID was achieved in 36.4%, 51.5%, and 66.7% of patients at the follow up on 4, 8, and 12 weeks, respectively. The differences among the groups in MCID were shown in **Table 3**.

**Table 3:** The differences of chi-square statistical outcome in percentage of MCID cases between each two groups.

groups	4-week follow-up		8-week follow-up		12-week follow-up	
	Chi-Square	P value	Chi-Square	P value	Chi-Square	P value
Group A vs. Group B	4.148	0.042*	5.195	0.023*	2.986	0.084
Group A vs. Group C	4.148	0.042*	10.288	<0.001*	16.542	<0.001*
Group B vs. Group C	–	–	0.978	0.323	6.066	0.014*

The data of percentages was compared by the Chi-squared test. *P<0.05, there were statistically significant difference.

The results of MCID differences within and between groups showed that the combination therapy was more beneficial than VED monotherapy at 12 weeks follow-up (*P*<0.001), and the power was 0.997 at a significance level of 0.05. Meanwhile, the combination therapy was more effective than Li-ESWT monotherapy at 12 weeks follow-up (*P*=0.014), although the power was 0.734 at a significance level of 0.05. The results of comparison within the combination therapy group showed that there were significant differences in MCID between 4 and 12 weeks of follow-up (*P*=0.014; **Table 4**), and the power was 0.824 at a significance level of 0.05.

**Table 4:** The differences of chi-square statistical outcome in percentage of MCID cases between each two various follow-up points.

Follow-up	Group A		Group B		Group C	
	Chi-Square	P value	Chi-Square	P value	Chi-Square	P value
4th week vs. 8th week	–	–	0.064	0.800	1.538	0.215
4th week vs. 12th week	0.108	0.742	–	–	6.066	0.014*
8th week vs. 12th week	0.108	0.742	0.064	0.800	1.567	0.211

The data of percentages was compared by the Chi-squared test. *P<0.05, there were statistically significant difference.

**Figure 1:**
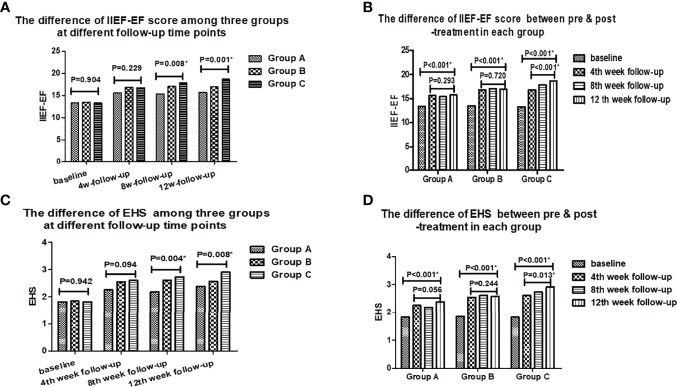
The differences of IIEF-EF and EHS scores among three groups at different follow-up time points and between pre & post-treatment in each group.

There were significant differences among there groups in the mean IIEF-EF scores and EHS, as well as SEP2 and GAQ1 at the 8th and 12th week follow-up. Additionally, there were significant differences in the average IIEF-EF scores and EHS between pre & post-treatment in each group (*P*<0.001), and at all follow-up time points in Group C (*P*=0.013), however, no differences were found at all follow-up time points in Group A and Group B (**Table 2** and **Figure 1**). There were no significant differences in SEP2, GAQ1 and GAQ2 among three groups at the 4th week follow-up, except for SEP3. Moreover, there were no remarkable differences in SEP3 and GAQ2 among three groups at the 8th and 12th week follow-up, and in the mean IIEF-EF scores and EHS at the 4th week follow-up.

### Comparison of Complications Among Three Groups

During treatment and follow-up, there were no moderate and severe penile pain or local ecchymosis cases in all patients. The 2 cases of mild pain and 1 case of mild local ecchymosis recovered without special management in each group. There were no marked differences in therapeutic complications among three groups.

## Discussion

Over the past few decades, accumulating evidences demonstrated that the occurrence and development of DMED possibly involved in multifactorial pathogenesis including metabolic, neurologic, vascular and muscular components (22–25). Recently, L-arginine, as an alternative treatment which is an essential substance for the synthesis of nitric oxide (NO), might be benefit for diabetic erectile dysfunction (26). Previous study indicated that there was a synergistic effect of the combination therapy L-Arginine plus tadalafil and combination therapy was superior to monotherapies (27). More and more combined therapeutic schemes had obtained satisfactory outcomes in patients with complicated ED of specific etiology, such as PDE5is combined with VED in the management of postprostatectomy erectile dysfunction (pPED) (28). Despite widespread use of combination therapy in clinical research and practice of ED (29), no published data are available concerning the efficacy of intensity extracorporeal shock wave combined with vacuum erectile device for patients with diabetic ED. We introduced the concept of combination therapy into the study to explore a more effective and safe treatment strategy by combination therapy of vacuum device, shockwaves and on demand oral PDE5is, for initial non-response to PDE5is diabetic patients with moderate erectile dysfunction. In this study, we found that monotherapy with VED or Li-ESWT might be have certain effects (in MICD) on moderate vascular DMED (17.6%, 36.4%, respectively). More importantly, combination therapy VED and Li-ESWT showed more effective than monotherapy in MICD (66.7%), as well as synergistic benefits in the short term. The potential mechanisms may be related to the underlying rehabilitation effect of vacuum erectile device in the prevention of cavernosal fibrosis and presence of promotion of low-intensity shock wave in penile nervous, vascular, and muscular tissue regeneration and improvement of endothelial function (30–32).

According to an initial study by Price et al. (33), 75% (33/44) of diabetic men with impotence were able to have satisfactory intercourse by vacuum tumescence therapy, which was regarded as an effective and simple treatment which required little investigation. In the other previous study on the combined therapy VED and PDE5is for ED by Chen et al. (34), thirty five men with ED who were ineffective for PDE5is in 80 cases firstly preferring PDE5i medication were treated with PDE5is combined with VED, and 26 patients of them were satisfied, that is, the treatment satisfaction rate increased from 56.3% to 88.8%. Besides, Canguven’s clinical research data showed that the mean IIEF-5 score in 69 men with ED caused by various reasons (including 16 patients with DMED) and poor responses to PDE5is increased significantly over baseline from 9.0 to 17.6 (*P*<0.001) after 4 weeks of combination therapy of VED and oral medication, and the results suggested that the combined therapy might be tried prior to initiating more invasive alternatives (35). Previous studies had indicated that vacuum constrictive devices (VCD) were usually reserved for patients who failed oral PDE5is, by improving hypoxia in corpus cavernosum, thereby inhibiting smooth muscle cell apoptosis and cavernous fibrosis (36, 37). In our study, VED was utilized for rehabilitative treatment without the use of tension rings, which was different from VCD with tension rings for the purpose of maintaining erection for successful sexual intercourse. However, based on our observations in this study, no more than 17.6% (yes%, MCID) of patients obtained certain curative effect during follow-up. It was our opinion that this relatively poor efficacy of vacuum therapy might be link with the major purpose of erectile tissue rehabilitation without the use of tension ring in the clinical trial. Our findings were kind of similar with the other study about VED by Raina (38), who assessed 109 patients with pPED and found that 17% of men had spontaneous erections sufficient for vaginal intercourse by the use of VED after 9 months, compared to 10% of men in the control group.

Li-ESWT, as an energy-based therapy technology, represents a new frontier of treatment geared towards reversing disease pathology rather than just treating symptoms (39). Li-ESWT might bring new hope to patients with multiple diabetic complications. Previous clinical trails showed that Li-ESWT had been tried to use for the management of diabetic complications such as diabetic foot ulcers and diabetic kidney disease (40, 41); and for the treatment of diabetic ED, and an increasing body of evidence demonstrated Li-ESWT was of satisfactory efficacy and fewer complications as a novel physical therapy of ED. Wang et al. reported that energy flux density (EFD) of 0.05mj/mm^2^ of Li-ESWT therapy could turn 71% (27/38) of PDE5is non-responders to responders and could improve erection hard enough for vaginal penetration at 16th week follow-up (42). In the other study reported by Tsai et al. (43), 67.3% of patients (35/52) could achieve an erection hard enough for intercourse under PDE5is medication at the 1-month follow-up after treatment of Li-ESWT and 63.5% (33/52) of patients could maintain the erectile function at the 3-month follow-up. These studies suggested that Li-ESWT could be regard as a salvage therapy for ED patients who failed to respond to PDE5is and initial severity of ED was the only significant predictor of a successful response. Our results showed that the mean IIEF-EF score and EHS were significantly higher at follow-up than those at baseline in Group B (*P*<0.05), additionally, the proportion of cases reaching MCID (39.4%) in Group B was obvious higher than those (14.7%) in Group A at 8th week follow-up, but no significant differences were found at 12th week follow-up between two groups (17.6% vs. 36.4%, *P*=0.084). The proportion of MCID obtained in this study was lower than that reported in the literature, which might be related to the initial severity of DMED. Out findings indicated that Li-EWST monotherapy was possibly more effective than VED monotherapy in the early stage, but the superiority of Li-EWST monotherapy to VED is relatively limited in improving response to PDE5is for moderate diabetic ED in the longer term.

Fortunately, the results of our study showed that 66.7% (yes%, MCID) of subjects received combined therapy of VED, Li-EWST and PDE5is achieved relatively higher efficacy than those in group A or group B at 12th week follow-up, which implied that there was the gradual emergence of synergistic effect between VED and Li-ESWT in the early stage. Current experiments showed that the potential mechanism of Li-ESWT for ED involved in improving endothelial function, penile progenitior cell recruitment and activation, as well as inhibiting apoptosis and atrophy of the corpus cavernosum (44–46). Assaly et al. (47) found that smooth muscle/collagen ratio increased 2.5-fold in spontaneously hypertensive rats (SHRs) received Li-ESWT compared with sham, whereas neuronal nitric oxide synthase (nNOS) was unchanged. However, Jeong et al.’s report (48) showed that ESWT could not only increase the expression of nNOS, but also enhanced the expression of α smooth muscle actin (αSMA), vascular endothelial growth factor (VEGF), platelet endothelial cell adhesion molecule-1 (PECAM-1) and phosphorylated endothelial nitric oxide synthase (P-eNOS) in the corpus cavernosum of DM rats, which was implied to benefit the recovery of the muscle, nerve and blood vessels of erectile tissues. Furthermore, Lin et al. (49) found that VED therapy could preserve penile size effectively in rats with bilateral cavernous nerve crush (BCNC) injuries by increasing cavernous blood oxygen saturation (SO_2_), and erection induced by VED was mainly due to the arterial blood inflow (62% arterial and 38% venous). Bosshardt et al.’s study (50) found that the average rigidity (monitored by Rigiscan) was >80% in 26 patients with ED after VED application and the increased penis volume was caused by 58% arterial and 42% venous inflow (calculated by blood gas analysis). In this study, combined therapy VED and Li-ESWT was more effective than either monotherpy. This finding suggested that regular VED physiotherapy could improve the blood supply of penile artery, which was similar to spontaneous nocturnal erection. In addition, sufficient penile length might improve their confidence in treatment, and provide good conditions in various place of penis for Li-ESWT positioning. Therefore, in addition to the subjects in Li-ESWT monotherapy, those patients in the other two groups received VED treatment during treatment and follow-up.

However, in the study, the patients with relatively severe and refractory DMED were selected, consequently, although the effectiveness of the combined group was acceptable, the overall effective rate of the subjects was low after treatment, and further treatment was insufficient after follow-up. Meanwhile, the small sample size and the short observation time limited the stronger persuasiveness of design concept of this study. An additional limitation is that the different type and exact dosage of PDE5is drugs and anti-diabetic drugs in different individuals during enrollment and follow-up, which might bring about the deflection in efficacy evaluation. The accurate diabetes time-course of individual patients was unavailable, which might also affect the results. Comparisons should be performed between different therapeutic regimen in our further studies, such as the combination therapy with LiESWT+ daily PDE5i. Moreover, the positive effects of the therapy on the penile vascularity should be evaluated by a Penile Doppler ultrasound, which could provide more objective efficacy indicators. Finally, as we known, the vascular damage of penile cavernous in non-diabetes patients was less than that in diabetes patients, therefore, this combined therapy would be more effective in patients with diabetes than in patients without diabetes. However, this problem needed to be explored by further studies with increasing sample size and type.

## Conclusion

Our findings showed the combined therapy Li-ESWT and VED was more beneficial to shift turn PDE5is nonresponders to responders for moderate impotence men with DM than Li-ESWT or VED monotherapy due to their synergistic effect. Moreover, this study provided evidences that patients with DMED who failed after taking oral PDE5i drugs and receiving VED or Li-ESWT could attempt to opt for an alternative physicotherapy (Li-ESWT or VED) prior to more invasive alternatives. The long-term efficacy and safety of this treatment remained to be further investigated in well-characterized patients by more multi-center, randomized, controlled trials.

## Data Availability Statement

The original contributions presented in the study are included in the article/**Supplementary Material**. Further inquiries can be directed to the corresponding authors.

## Ethics Statement

The studies involving human participants were reviewed and approved by Nanjing Jiangning hospital ethics committee. The patients/participants provided their written informed consent to participate in this study. Written informed consent was obtained from the individual(s) for the publication of any potentially identifiable images or data included in this article.

## Author Contributions

RT, JC, JY, and QT designed the experiments. RT, DW, YL, LX, JJ, JW, SZ, CJ, JL, and QT contributed to clinical data collection and assessment. RT, JC, JX, JY, and QT analyzed the results. RT, JC, JY, and QT wrote the manuscript. All authors read and approved the final manuscript.

## Funding

This work was supported by the key project of scientific research development fund of Kangda College of Nanjing Medical University in China (No. KD2019KYJJZD020); General project of Natural Science Foundation of Xinjiang Uygur Autonomous Region (2022D01A23).

## Conflict of Interest

The authors declare that the research was conducted in the absence of any commercial or financial relationships that could be construed as a potential conflict of interest.

## Publisher’s Note

All claims expressed in this article are solely those of the authors and do not necessarily represent those of their affiliated organizations, or those of the publisher, the editors and the reviewers. Any product that may be evaluated in this article, or claim that may be made by its manufacturer, is not guaranteed or endorsed by the publisher.
