# Supplementary figures and images for: The Efficacy of Li-ESWT Combined With VED in Diabetic ED Patients Unresponsive to PDE5is: A Single-Center, Randomized Clinical Trial
Source: Front Endocrinol (Lausanne). 2022 Jun 23;13:937958. doi: 10.3389/fendo.2022.937958 (PMC9259797; doi:10.3389/fendo.2022.937958)

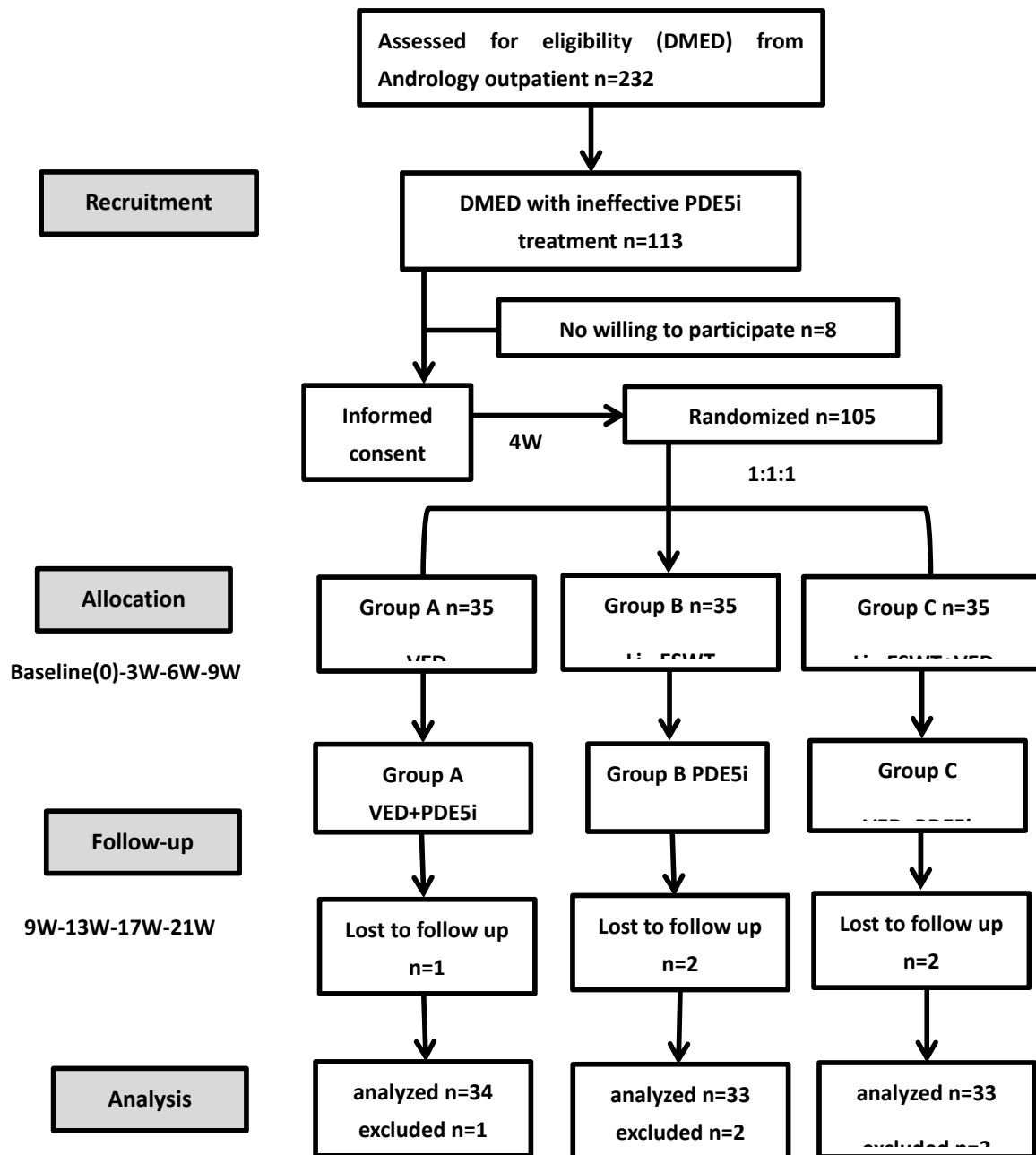

Supplementary Figure 1 The design of this study

Supplement: Supplementary file 1 [file Image_1.pdf]
